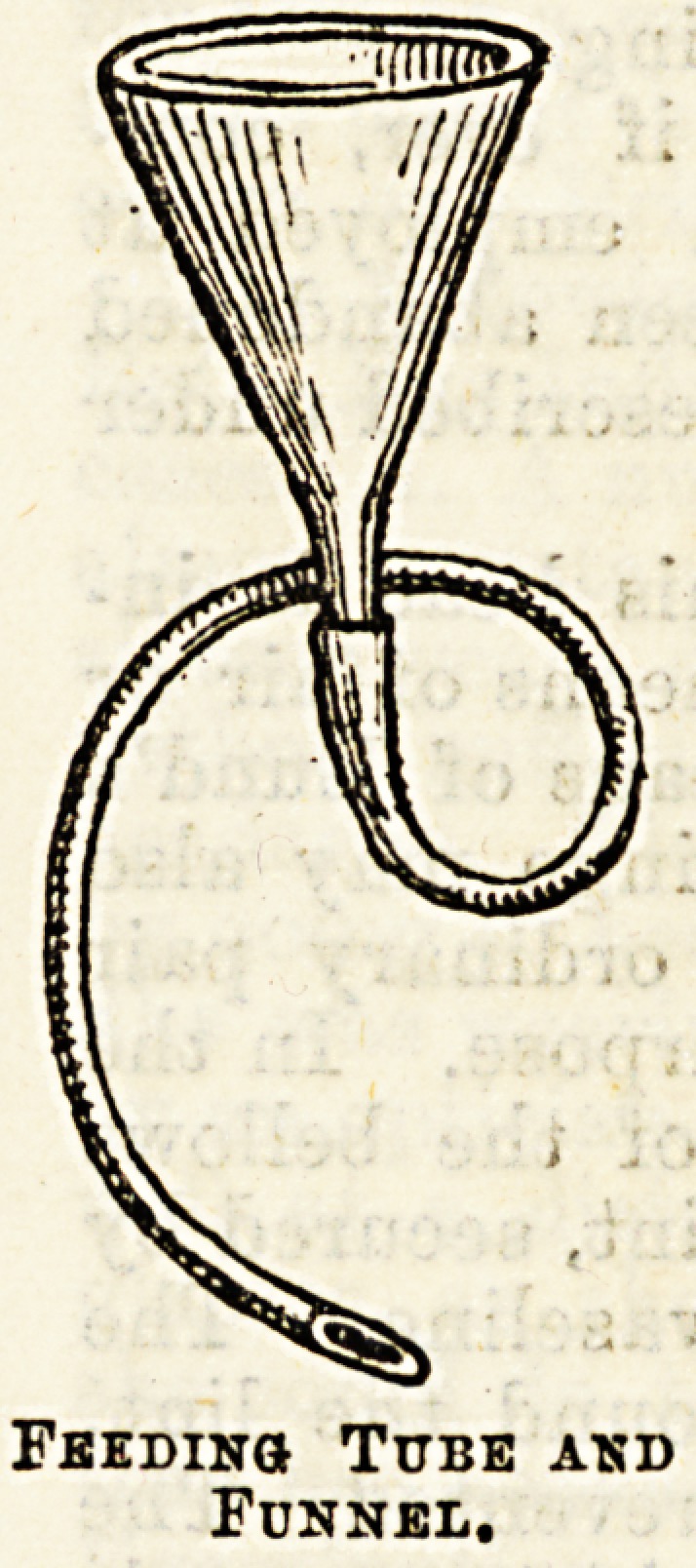# The Treatment of Chorea

**Published:** 1893-03-18

**Authors:** 


### The Hospital Clinic.

[*The Editor will be glad to receive offers of co-operation and contributions from members of the profession All letters should be addressed to* The Editor, The Lodge, Porchester Square, London, W.]

### LONDON HOSPITAL.

### The Treatment of Chorea.

The various modes of treatment of chorea as carried out at this hospital have as a common basis the idea of securing for the patient as complete rest as possible; rest of the mind as well as rest of the body. In the methodical routine of a hospital ward, with its fainter personal interests, the small everyday worries of home life are soon lost in the background, and a condition of greater mental quiet secured.

The ordinary cases of chorea of childhood and youth are for practical purposes divided into the rheumatic and non-rheumatic; in the former, special treatment directed to the rheumatic state has been found to be of benefit, small doses of salicylate of sodium or salicine being given in addition to the general treatment applicable in all cases of chorea.

In all cases complete rest in bed is at first strictly enjoined, with as much food as the patient can eat. The Weir-Mitchell treatment is used in some cases with conspicuous success. To the complete rest and absence of mental strain a forced diet is added, the patient being encouraged to eat a much larger quantity of food than would be required to sustain life, and for this purpose is fed frequently, usually at intervals of about two hours. If sleep at night is disturbed by the constancy of the movements, alcohol, either as whiskey or brandy, is given at bed-hour, with the result that sounder and more beneficial sleep is obtained. Daily, for from a quarter to half-an-hour, the patient is massaged. Care is also taken to keep the bowels acting freely, either with saline purgatives or other simple laxatives. Under this treatment the anæmia, so often present with the chorea, improves, and as the patient gains in weight the movements lessen and disappear. In slight cases rest in bed, with ordinary nutritious food, is sufficient to effect a cure, but this is hastened by the addition of tonics in different forms. Iron is frequently given, either as the sulphate in the form of pill with or without aloes, or as the carbonate or wine, either by itself or in conjunction with cod liver oil or malt extract. Another tonic found to be of great service is arsenic, given as small doses of Fowler's solution. Arsenic, however, is found in some cases to have a direct effect on the course of the disease, quite apart from its tonic action. For this purpose it is given in constantly increasing doses of Fowler's solution till some slight symptoms of intolerance are produced, or a maximum dose of ten to fifteen minims is reached. Other drugs, such as zinc oxide or sulphate, and strychnine have been used, but experience has not shown that they have other than a merely tonic effect.

Belladonna has been found in a few cases to have a distinct effect in lessening the movements after other drugs have failed, though, as a rule, it has not proved itself to be of much use. A similar conclusion has been arrived at with regard to the use of quinine in moderate doses, markedly useful in a few, useless except as a tonic in the great majority of cases.

In conjunction with other treatment, cold spinal douches are used once or twice a day, and seem to have a great effect in lessening the movements. The patient stands in a foot-bath containing warm water, the spinal column is sponged down for about half a minute to a minute with cold water, quickly dried, and sent back to bed. The movements for an hour or so after are distinctly less, the patient frequently going to sleep. The ether spray has been used as a substitute for the douche, but is by no means as efficient.

In the most severe cases, where the movements are so severe as to prevent eating or sleep, sedatives have to be used to procure sleep, and render feeding possible. For this purpose chloroform is administered, and continued even for thirty-six or forty-eight hours in the worst cases, the administration being, as a rule, intermittent, alternating with full doses of chloral, morphia, bromide of potash, or ammonium, the three last not having been found anything like as useful as chloral.

Feeding Tube and Funnel.

While rest is thus ensured, food is given by a stomach tube, a soft rubber catheter attached to a four or six-ounce glass funnel being used. The tube, smeared with glycerine, is passed into the stomach, either through the mouth or along the floor of the nose. Liquid nourishment, such as milk or beef-tea, with meat extract, eggs, and brandy, being thus administered every four hours, till the movements have diminished sufficiently to allow food to be taken by the mouth. In these, as in every case of chorea, the greatest care is taken to ensure regular action of the bowels.

Hot packing has been found very useful as a sedative, and often gives several hours' quiet sleep without the administration of narcotics.

The patient being stripped lies on the bed and is wrapped from neck to feet in a sheet wrung out of hot water, two or three blankets outside this, and a mackintosh underneath to preserve the bed. The patient remains in this for from half to two or three hours, if comfortable, is taken out, dried quickly, and put to bed. Several hours' quiet sleep usually follow.

When the movements are excessive care has to be taken to prevent the formation of bedsores from the constant movements; either a water-bed or cotton wool on the limbs prevent this. To keep the patients from tumbling out of bed is sometimes a difficulty; this is done by having a long strip of canvas or sheeting about eighteen inches deep attached to the head of the bed, brought down one side round a support at the bottom, and up the other side of the bed to be again tied to the head. The lower border is attached by tapes, either to the side irons of the bed-frame, or to the mattress; and the free upper border being about a foot higher than the level of the bed, turns it into a large cot with sides, and renders it almost impossible for the patient to fall out.

## Figures and Tables

**Figure f1:**